# Trajectory of frailty and association with adverse outcomes in patients with end-stage kidney disease over the first year of dialysis

**DOI:** 10.1038/s41598-025-18961-4

**Published:** 2025-10-08

**Authors:** Kerry-Lee Rosenberg, Aegida Neradova, Ioannis Kostakis, Heidy Hendra, David Wright, Aine Burns, Andrew Davenport

**Affiliations:** 1 Department of Renal Medicine, University College London, London, UK; 2 Amsterdam UMC, Dianet Amsterdam, Amsterdam, The Netherlands; 3 Amsterdam UMC, Amsterdam, The Netherlands; 4 Royal Free London NHS Foundation Trust, London, UK

**Keywords:** End-stage kidney disease, Frailty, Haemodialysis, Peritoneal dialysis, Older adults, Outcomes research, Nephrology, Renal replacement therapy, Geriatrics, Prognosis

## Abstract

Little is known about trajectory of frailty as patients with advanced chronic kidney disease transition onto dialysis. This retrospective cohort study aims to describe change in frailty from dialysis initiation over the course of the first year of treatment. Secondly, we aim to examine the association of frailty and change in frailty over time with mortality and hospitilisation. The study included incident haemodialysis or peritoneal dialysis patients. Clinical frailty scale (CFS) was recorded at dialysis start and after 6 and 12 months of treatment. Multilevel mixed effects linear regression models were used to examine change in CFS from baseline to 6 and 12 months after start of dialysis. Kruskal-Wallis tests were used to assess the association between CFS with number of hospitilisations. Cox proportional hazard models were used to examine the association of CFS with survival time after dialysis initiation. The study included 293 patients, 65% haemodialysis, 35% peritoneal dialysis. Characteristics of the patients on each modality were similar. Mean CFS pre-dialysis was 4.0 (95% CI 3.81–4.21). Time on dialysis was associated with a mean increase in CFS of 0.18 per 6 months (95% CI 0.06 to 0.31), independent of confounding variables. Frailty prior to initiation of dialysis (defined as CFS ≥ 5) was associated with increased hospitilisations, compared to the non-frail group (*p* = 0.011). An increase in frailty score between pre-dialysis and 6 months was associated with increased risk of mortality (HR 1.35, 95% CI 1.01–1.80). Our findings highlight the importance of pre-dialysis frailty screening in order to identify at-risk patients and inform prognosis and patient counselling. Frailty trajectory in the first 6 months of dialysis treatment is a predictor of adverse outcomes.

**Supplementary Information:**

The online version contains supplementary material available at 10.1038/s41598-025-18961-4.

## Introduction

Kidney replacement therapy (KRT) for end stage kidney disease (ESKD) is a worldwide health challenge, with prevalence estimated at 3.9 million people in 2017^1^. This number is increasing^1^ and is associated with significant morbidity, mortality and economic burden^2,3^. Frailty is defined as a syndrome of cumulative decline across multiple physiological systems, resulting in decreased reserve and increased vulnerability to adverse outcomes^4^. Frailty has been associated with increased mortality and rates of hospitilisation in the dialysis population^5–10^. It is not limited to the older ESKD patient, but commonly reported in both younger and older patients receiving KRT^5,6^.

Despite the clinical impact of frailty, little is known about its likely trajectory as patients with advanced chronic kidney disease (CKD) transition on to KRT. One prospective study has reported no overall improvement or decline in frailty status for patients already started on haemodialysis (HD)^11^. Conversely, worsening frailty has been reported in patients established on peritoneal dialysis (PD)^12^. Neither of these studies included patients with pre-dialysis CKD and further evidence is required to better understand the progression of frailty after start of KRT.

This study aims firstly to describe the trajectory of frailty as patients with advanced CKD transition on to KRT and over the course of the first year of treatment and to compare change in frailty between those receiving HD versus PD. Secondly, we aim to examine the association of frailty and change in frailty score over time with mortality and hospitilisation.

## Methods

### Settings and data collection

This retrospective, observational cohort study was undertaken in a major tertiary university hospital, located in North Central London, serving a large and ethnically diverse urban population.

The electronic patient recorded was used to identify all adult patients, who were newly started on maintenance HD or PD for ESKD between 1 January 2016 and 1 January 2020. Patients who received a kidney transplant were excluded. Frailty status as described by the Rockwood Clinical Frailty Scale is recorded at our center by both nephrologists and renal nurses as part of routine clinical care^13^. Staff receive training in using the CFS and the clinical records system provides a prompt with the definitions of each point on the scale. Those patients who had a clinical frailty score (CFS)^13^ recorded within 6 months prior to and/or 6 to 12 months after the initiation of KRT were selected for analysis.

Additional parameters recorded included age, sex, ethnicity, primary renal disease, body mass index (BMI), dialysis modality and any change in modality. Charlson comorbidity index^14^, both with age adjustment (aCCI) and without (CCI), was calculated for each patient. Date of death was recorded. In addition, the number of hospitilisations at our centre and associate centre (Royal Free Hospital and Barnet Hospital, which share an electronic patient records system) were also recorded.

### Outcomes

The primary outcome was change in frailty score from baseline (i.e. 0 to 6 months before initiation of KRT) to 6 and 12 months after initiation of dialysis. The secondary outcomes were association of frailty score with number of hospitilisations, association of change in frailty score over time with hospitalization and, finally, association of frailty and change in frailty score with survival after initiation of KRT.

### Statistical analysis

Statistical analysis was carried out using Stata 17. A significance level of 5% was applied throughout. A descriptive analysis of the study cohort was carried out and the characteristics of those on HD and PD were compared. Continuous variables with a normal distribution were compared using the mean and standard deviation (SD). Those with non-normal distribution were compared using the median and interquartile range (IQR). CFS was treated as a continuous variable.

Multilevel mixed effects linear regression models were used to estimate change in frailty score from baseline to 6 and 12 months after start of KRT. The model was repeated with adjustment for potential confounders including sex, dialysis modality, baseline frailty score and CCI. Results were expressed as mean change in CFS per 6 months on dialysis, with a 95% confidence interval (95% CI).

The amount of missing frailty data at each time point was explored and the characteristics of those with complete data (CFS recorded at all three time points) compared to those without. Multiple imputation was used as a sensitivity analysis in order to check the robustness of the results. The analysis was repeated using different numbers of imputations and point estimates and standard errors compared. The imputation model included age, sex, CCI and dialysis modality.

Spearman’s rank correlation test was used to assess the relationship between frailty score at each of the 3 time points with number of hospitilisations per month of follow up time. Results were reported as a correlation coefficient (r) and p-value.

The cohort was then grouped according to frailty status (frail versus non-frail) before dialysis. Presence of frailty was defined as a score of 5 or higher^13^. The mean number of admissions per month was compared between the groups using a Kruskal-Wallis test. This was repeated at 6- and 12-months post-dialysis. The analysis was also repeated at all three time points using different cut-off scores (CFS ≥ 6 and CFS ≥ 7). Finally, the cohort was grouped according to the change in frailty score from pre-dialysis to 6 months (improved CFS, static CFS and worsening CFS) and the mean number of admissions once again compared. This was repeated using change in score from pre-dialysis to 12 months.

Median survival after dialysis initiation was compared by baseline CFS and by change in CFS from baseline to 6 months (grouped as those with improved CFS, static CFS or worsening CFS). A cox proportional hazards model was used to assess survival after the initiation of KRT. Frailty score at 6 months post start of dialysis and change in frailty score from pre-dialysis to 6 months were modelled against sex, dialysis modality, any switch in modality, BMI, CCI and primary renal disease. Results were reported as a hazard ratio (HR) and 95% CI.

### Ethics

This retrospective audit followed and complied with United Kingdom (UK) National Research Ethics Services (NRES) clinical audit and service development, with the audit of clinical service registered and individual patient consent waived by NRES. In accordance with NRES guidelines data patient data was appropriately anonymised. Health Research Authority approval was confirmed (IRAS ID 282462).

## Results

### Patient characteristics

The study included 293 patients. Baseline characteristics of the cohort are summarized in Table 1. The median age at initiation of KRT was 68 years (IQR 56 to 76) and 64.9% were male. In terms of ethnicity, the cohort was 55.6% white, 15% Black, 10.2% South Asian, 1% other ethnic groups and 18.1% unknown ethnicity. The number of patients treated with hemodialysis was 191 (65%) and 102 (35%) with peritoneal dialysis. There was no significant difference in baseline estimated glomerular filtration rate (eGFR), age, ethnicity, CCI or baseline frailty score between patients subsequently treated by the two modalities. The haemodialysis group included a higher proportion of people with diabetes. Median follow up time was 18 months (IQR 11 to 25 months).

**Table 1:** Baseline characteristics of the study cohort.

	All (*n* = 293)	Haemodialysis (*n* = 191)	Peritoneal dialysis (*n* = 102)	*p*-value (haemodialysis vs. peritoneal dialysis)
Age at dialysis start (years); median (IQR)	68 (56–76)	68 (57–75)	67 (55–77)	*p* = 0.84^1^
Male sex; n (%)	190 (64.9)	128 (67.0)	62 (60.8)	*p* = 0.287^2^
eGFR at dialysis initiation^3^; median (IQR)	6.3 (5.0–7.8)	6.4 (4.9–7.9)	6.0 (5.2–7.6)	*p* = 0.819^1^
Ethnicity ; n (%)				
White	163 (55.6)	111 (58.1)	53 (51)	*p* = 0.788^2^
Black	44 (15)	26 (13.6)	18 (17.7)	
South Asian	30 (10.2)	18 (9.4)	12 (11.8)	
Other ethnic group	3 (1)	2 (1.1)	1 (0.9)	
Unknown	53 (18.1)	34 (17.8)	19 (18.6)	
Primary renal disease; n (%)				
Cystic kidney disease	15 (5.1)	8 (4.2)	7 (6.9)	*p* = 0.293^2^
Pyelonephritis/obstruction	17 (5.8)	12 (6.3)	5 (4.9)	
Glomerulonephritis	30 (10.2)	16 (8.4)	14 (13.7)	
Renovascular disease/ hypertension	36 (12.3)	20 (10.5)	16 (15.7)	
Diabetic kidney disease	138 (47.1)	94 (49.2)	44 (43.1)	
Other causes	57 (19.5)	41 (21.5)	16 (15.7)	
Comorbidities; n (%)				
Diabetes mellitus	178 (60.8)	124 (65)	54 (52.9)	*p* = 0.045^2^
Ischaemic heart disease/ left ventricular failure	81 (27.7)	57 (29.8)	24 (23.5)	*p* = 0.250^2^
Stroke/ TIA	29 (9.9)	22 (11.5)	7 (6.9)	*p* = 0.204^2^
Peripheral arterial disease	23 (7.9)	15 (7.9)	8 (7.8)	*p* = 0.998^2^
Charlson-comorbidity score; mean (SD)	3.9 (1.5)	4.0 (1.5)	3.8 (1.4)	*p* = 0.173^4^
Age-adjusted Charlson-comorbidity score; mean (SD)	6.1 (2.1)	6.2 (2.2)	5.8 (2.0)	*p* = 0.1095^4^
Baseline frailty score; mean (SD)^**5**^	4.1 (1.5)	4.2 (1.5)	3.8 (1.5)	*p* = 0.1004^4^
Time of baseline CFS recording (number of days prior to dialysis start); median (IQR)^5^	50 (25–112)	53 (25–113)	48.5 (27–84)	*p* = 0.544^1^

1 Kruskal Wallis p-value.

2 Chi-squared p-value.

3 Estimated glomerular filtration rate calculated using the CKD-Epi 2021 equation.

4 Unpaired t-test p-value.

5 *n* = 189 (104 missing values).

### Prevalence of frailty

**Fig. 1:**
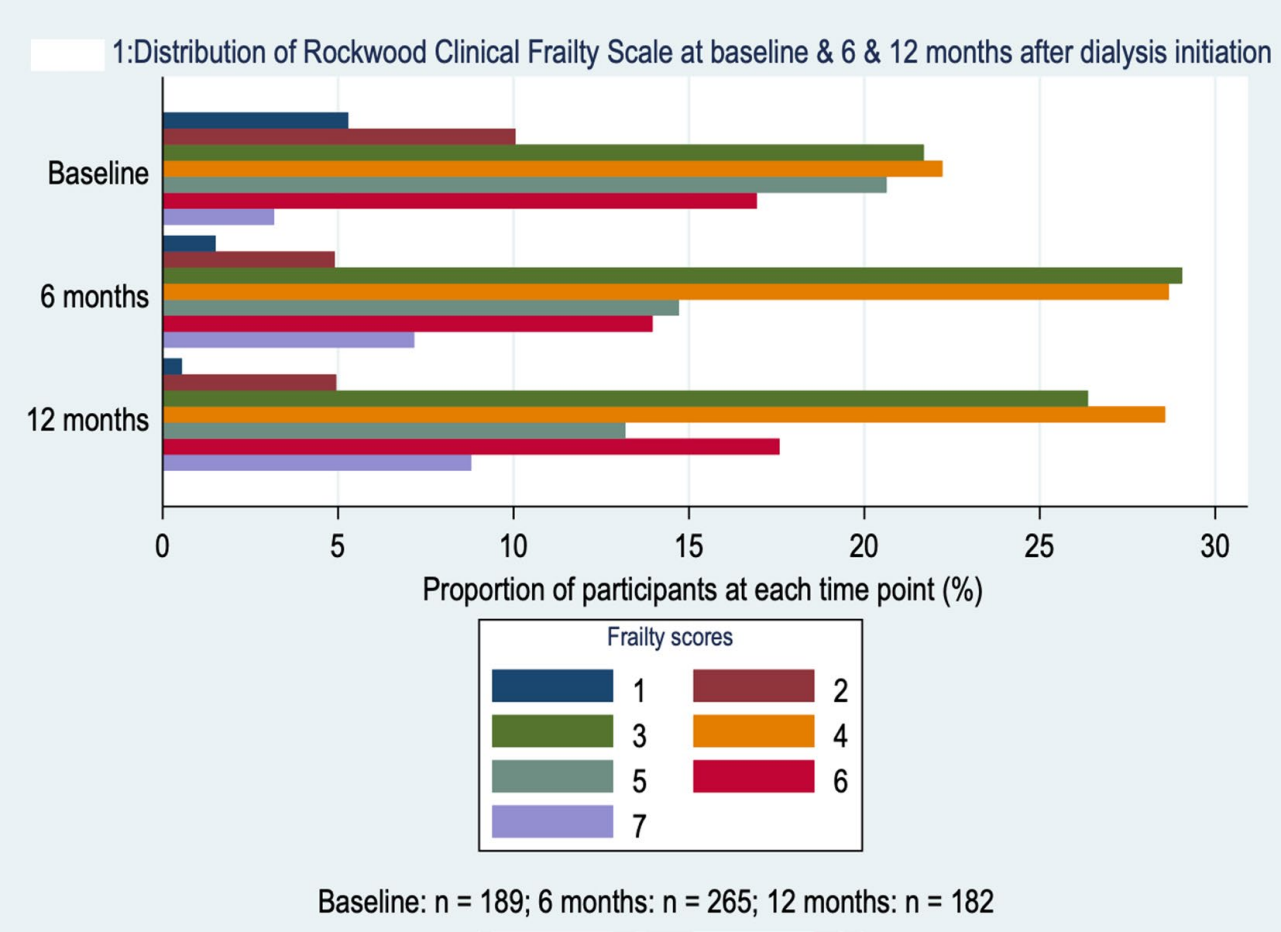
Distribution of Rockwood clinical frailty scale at baseline & 6 & 12 months after dialysis initiation.

At baseline, 189 patients had a CFS recorded and 77 (40.7%) were categorized as frail (CFS of 5 or higher). At 6 months after dialysis start, 265 patients had a CFS recorded and 95 (35.9%) were frail. At 12 months, CFS was recorded in 182 patients and 72 (39.6%) were frail. Figure 1 shows the distribution of frailty scores at each time point.

Baseline CFS increased with age, from 3.63 (SD 1.84) in those < 55 years to 4.38 (SD 1.33) in those ≥ 75 (supplementary Table 1); albeit the difference between the groups did not meet the threshold for significance (*p* = 0.087).

### Change in frailty score

In total, 164 patients had a frailty score measured at baseline and at 6 months. CFS remained the same in 78 patients (48%) and decreased in 23 (14%). CFS increased (i.e. worsened) in 63 patients (38%). There was no difference in age, sex, ethnicity, CCI, dialysis modality or presence of comorbidities between those in whom CFS stayed the same or decreased and those in whom CFS increased. In total, 120 patients had a frailty score measured at baseline and at 12 months, and the CFS remained the same in 29 patients (24%), decreased in 39 (33%) and increased in 52 (43%). As shown in Table 2, the group with worsening frailty scores was younger than those with stable or improved frailty scores. This group also had lower CCI, albeit the p-value for comparison of means was marginally above the threshold for significance (*p* = 0.051). There was no difference between the groups in sex, ethnicity, presence of comorbidities or dialysis modality.

**Table 2:** Characteristics of group with an increase in frailty score from baseline to 12 months, compared to those with no change or a decrease in score (*n* = 120).

	Increase in frailty score (*n* = 52)	Decreased or stable frailty score (*n* = 68)	*p*-value
Age at dialysis start (years); median (IQR)	67.5 (51.5–74.5)	74 (67–80)	*p* < 0.001^1^
Male sex; n (%)	37 (71.2)	47 (69.1)	*p* = 0.809^2^
Dialysis modality; n (%)			
Haemodialysis	37 (71.2)	48 (70.6)	*p* = 0.946^2^
Peritoneal dialysis	15 (28.9)	20 (29.4)	
Charlson-comorbidity score; mean (SD)	3.7 (1.4)	4.2 (1.5)	*p* = 0.051^3^
Comorbidities; n (%)			
Diabetes mellitus	29 (55.8)	47 (69.1)	*p* = 0.133^2^
Ischaemic heart disease/ left ventricular failure	12 (23.1)	23 (33.8)	*p* = 0.199^2^
Stroke/ TIA	5 (9.6)	9 (13.2)	*p* = 0.540^2^
Peripheral arterial disease	3 (5.8)	5 (7.5)	*p* = 0.730

1 Kruskal-Wallis p-value.

2 Chi-squared p-value.

3 One way ANOVA p-value.

In a mixed effects model, including a random intercept for the individual (*n* = 293), mean CFS pre-dialysis was 4.0 (95% CI 3.81–4.21), compared to 4.2 (95% CI 4.03–4.39) at 6 months after start of KRT and 4.4 (95% CI 4.18–4.58) at 12 months after KRT. A Wald test for comparison of means showed a significant difference in CFS across the time points (*p* = 0.014). There was a mean increase in CFS of 0.18 per 6 months on KRT (95% CI 0.06 to 0.31). The association between time on dialysis and change in CFS persisted after adjustment for confounders; including age, sex, CCI, dialysis modality and baseline frailty score. The crude and adjusted models are shown in Table 3.

**Table 3:** Mixed effects linear regression model for the association of mean clinical frailty score (CFS) with time on dialysis.

	Unadjusted model (*n* = 293)		Model adjusted for age, sex, Charlson comorbidity index (CCI) and dialysis modality (*n* = 293)		Model adjusted for age, sex, CCI, dialysis modality and baseline frailty (*n* = 189)	
	Effect estimate	95% confidence interval	Effect estimate	5% confidence interval	Effect estimate	95% confidence interval
Mean change in CFS per 6 months on dialysis	0.18	0.06–0.31	0.21	0.09–0.33	0.16	0.03–0.28

**Fig. 2:**
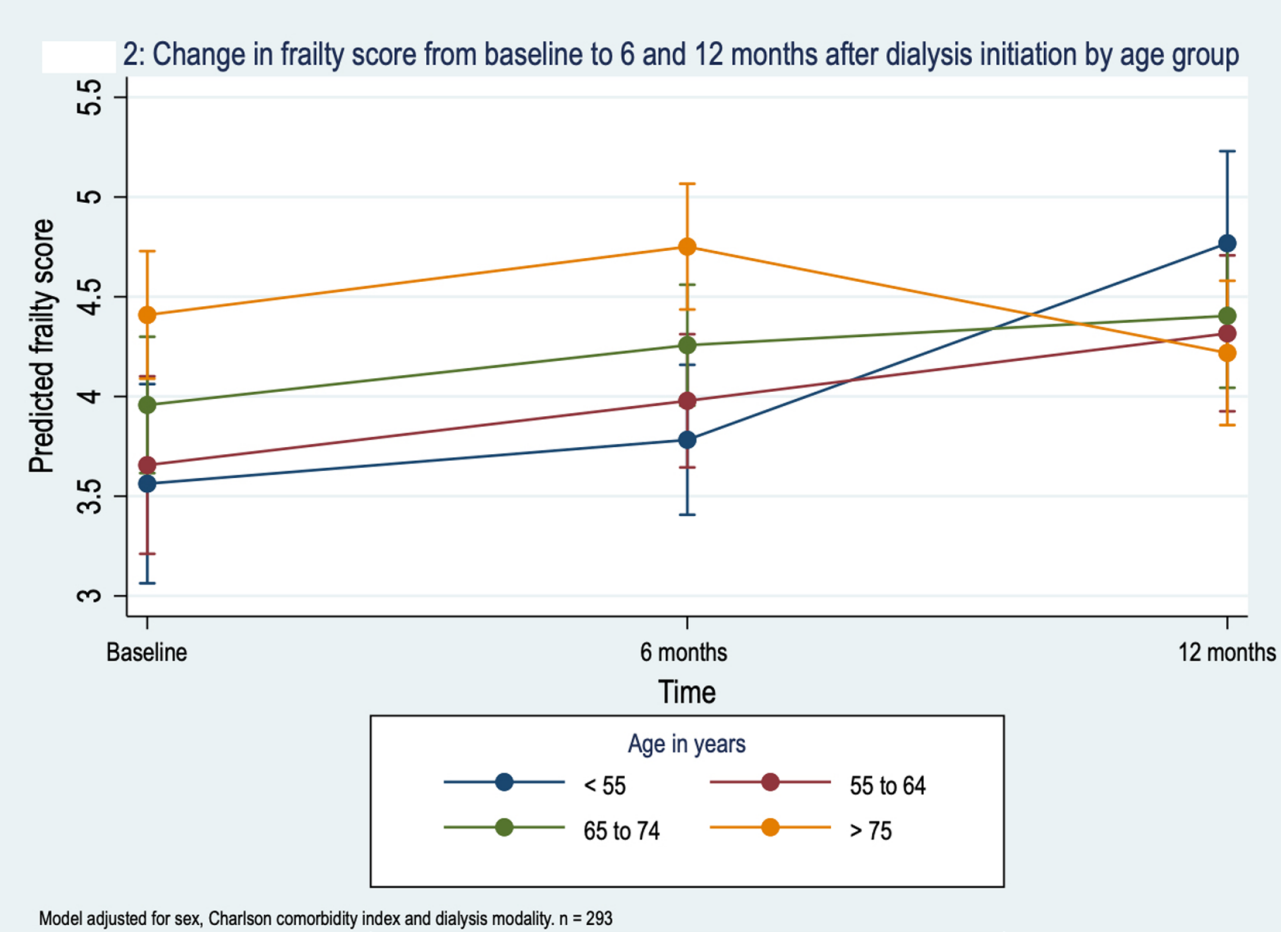
Change in frailty score from baseline to 6 and 12 months after dialysis initiation by age group.

The association of time after starting dialysis and change in CFS was not modified by CCI, dialysis modality or presence of comorbidities. There was, however, evidence of effect modification by age (Table 4; Fig. 2). CFS increased over time in all age groups, with the exception of those over 75 in which there was no change.

**Table 4:** Effect modification of the association of mean clinical frailty score (CFS) with time on Dialysis by age (*n* = 293).

Age in years	Mean change in CFS per 6 months on dialysis	95% Confidence interval	Wald test for effect modification
< 55	0.6	0.26–0.95	Chi^2^ = 11.4; *p* = 0.001
55 to 64	0.37	0.07 – 0.68	
65 to 74	0.28	0.03–0.54	
≥ 75	-0.07	-0.032–0.17	

1 Model adjusted for sex, Charlson Comorbidity index and dialysis modality.

In total, 105 patients had complete data; with CFS recorded at all three time points. This group were older than those with CFS recorded at only one or two time points (*p* < 0.001) but there were no significant differences by sex, ethnicity, dialysis modality or mean CCI. Mean CFS at each timepoint was similar between those with and without complete data across all time points. The proportion of missing observations across the three time points was 27.8%. Multiple imputation analysis using 10, 40 and 80 imputations showed similar effect estimates to one another and the principle data (Supplementary Table 2).

### Association of frailty with hospitilisations

We report a positive correlation between number of hospitilisations and CFS prior to initiation of KRT (*r* = 0.133, *p* = 0.046) and between hospitilisations and CFS 6 months after start of KRT (*r* = 0.142, *p* = 0.02). There was no significant correlation between hospitilisations and CFS at 12 months after the start of KRT. In addition, an increase in CFS from pre-dialysis to 6 months post starting and pre-dialysis and 12 months post were both positively correlated with number of hospitilisations (*r* = 0.210, *p* = 0.007 and *r* = 0.225, *p* = 0.023 respectively).

Number of hospitilisations per month was larger amongst those with frailty (CFS ≥5) at baseline compared to the non-frail group (*p* = 0.011). Amongst those with frailty at baseline, there was no difference in hospitilisation between those receiving haemodialysis and those on PD. At 6 months after the initiation of KRT, there was no difference in number of hospital admissions between those with frailty of any degree (CFS ≥5) and the non-frail group. However, number of admissions was higher in the group with CFS ≥ 6 (moderate frailty and higher), compared to those with CFS < 6. At 12 months, there was no difference in number of admissions at any frailty score cut off.

Finally, the number of hospitilisations per month was higher amongst those who had an in increase in CFS between baseline and 6 months compared to those with stable or improved CFS (*p* = 0.05).

### Association of frailty with survival after initiation of KRT

**Fig. 3:**
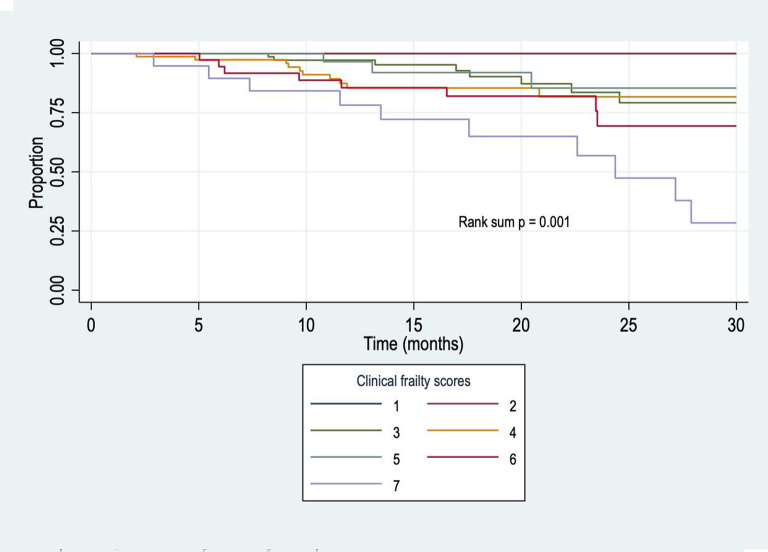
Kaplan-Meier curve for dialysis initiation by Rockwod clinical frailty scale at 6 months after dialysis start (n=265).

Median survival did not differ by CFS before commencing KRT. Median survival for those with a CFS of 7 at 6 months post initiation of KRT was 24 months (Fig. 3). However, median survival had not yet been reached in any other CFS group (i.e. fewer than 50% of patients had died amongst groups with CFS of 1 to 6) (rank sum *p* = 0.001).

**Fig. 4:**
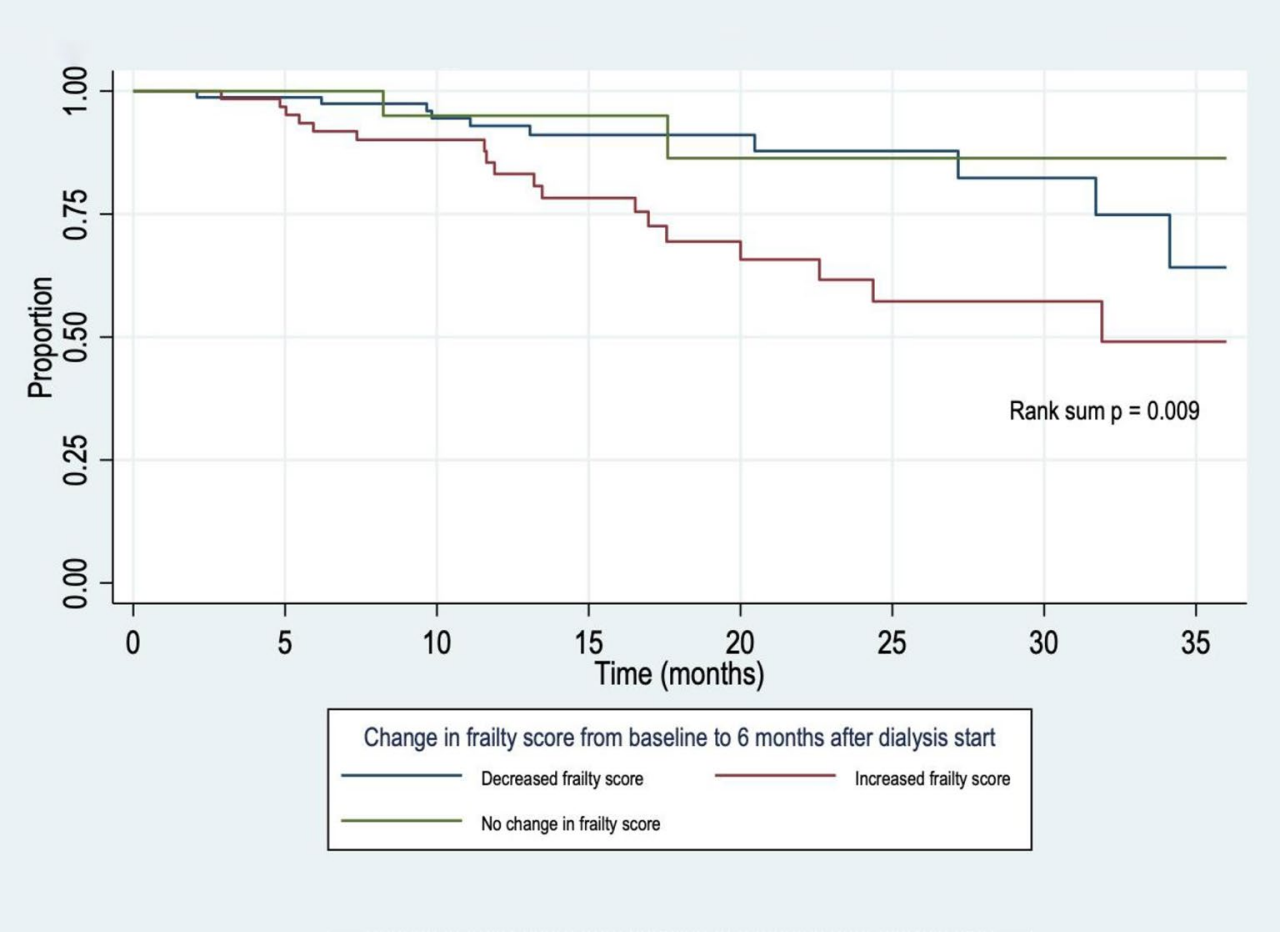
Kaplan-Meier survival curve showing survival after dialysis start by change in frailty score from baseline to 6 months (n=164).

Median survival was 31 months amongst those with an increase in CFS from baseline to 6 months after dialysis (Fig. 4), whilst median survival had not yet been reached for those with a stable or decreasing score (rank sum *p* = 0.009).

A cox proportional hazards model demonstrated no association between baseline frailty score and mortality. However, higher frailty score at 6 months was associated with an increased risk of death after initiation of KRT in a fully adjusted model (HR 1.54, 95% CI 1.22–1.92). In addition, an increase in frailty score between pre-dialysis and 6 months was associated with an increased risk of mortality (HR 1.35, 95% CI 1.01–1.80). These models are shown in Table 5.

**Table 5:** Cox regression models for the association of frailty scores at baseline and 6 months and change in frailty score with survival after Dialysis initiation. Results shown as hazard ratios and 95% confidence interval.

	Model 1^1^	Model 2^1^ (*n* = 265)	Model 3^2^ (*n* = 265)	Model 4^1^ (*n* = 164)	Model 5^2^ (*n* – 164)
Baseline frailty score at dialysis start	1.13 (0.91–1.40)	-	-	-	-
Frailty score at 6 months after dialysis start	-	1.47 (1.20–1.80)	1.54 (1.22–1.92)	-	-
Change in frailty from baseline to 6 months (per in unit increase in frailty score)	-	-	-	1.36 (1.03–1.81)	1.35 (1.01–1.8)

1 Unadjusted model.

2 Adjusted for age, sex, dialysis modality and Charlson comorbidity index.

## Discussion

Our findings demonstrate that there is no overall improvement in frailty status as patients with advanced CKD transition onto dialysis and in the first year of treatment. Patients with an increase in frailty score over the first 12 months of treatment were younger than those with stable or improved frailty score, but otherwise had similar characteristics. Time on dialysis was associated with an increase in frailty and this association was independent of age, sex, ethnicity, dialysis modality and burden of comorbidities. Furthermore, in this large cohort of hemodialysis and peritoneal dialysis patients with comparable baseline characteristics, there was no difference in frailty trajectory between the two modalities. Frailty trajectory was modified by age, with no change in frailty over time observed in those of 75 or older. Frailty prior to dialysis (CFS ≥ 5 at baseline) was associated with an increased number of hospitilisations. In addition, a deterioration in frailty status from pre-dialysis to the 6-month mark was associated with a both an increased number of hospitilisations and risk of death, compared to those with static or improved frailty scores.

The prevalence of frailty in our study was comparable to previously described dialysis cohorts^15,16^. Previous studies have described a higher prevalence of frailty amongst those with CKD in comparison to patients with normal kidney function^17^ and an association between declining estimated glomerular filtration rate (eGFR) and increasing frailty^18,19^. Evidence examining the trajectory of frailty status before and after initiation of KRT, however, is limited and conflicting. Johansen et al. reported no overall change in the prevalence of frailty over the course of 24 months in a cohort of 762 patients, already established on haemodialysis^11^. In contrast, a prospective study in a smaller cohort of 94 patients treated with peritoneal dialysis reported increasing frailty over a mean follow up period of 45 months^12^. Both studies included only prevalent dialysis patients. Our study adds to the existing body of evidence by examining the change in frailty status as patients with advanced CKD transition onto KRT, as well as over the course of the first year of treatment. A study by Tamura et al. reported on incident dialysis patients resident in nursing homes and observed a sharp decline in functional status after initiation of KRT^20^. The increase in frailty in our cohort was comparatively modest. The participants in the study by Tamura et al. were older (73.4 years) than our cohort (68 years) and the prevalence of congestive heart failure was significantly higher (66% versus 9.9%). Also, as nursing home residents they may have been more functionally dependent at baseline than our cohort. In our study patients over 75 years of age had no change in frailty status after initiation of dialysis, despite higher baseline frailty scores. It is possible that this finding reflects the shared-decision making process and careful selection of older patients who are started on haemodialysis in our center, as well as successful multidisciplinary care once established on dialysis.

Our study is one of the first to directly compare frailty trajectory in patients starting on hemodialysis compared with peritoneal dialysis. There was no difference in baseline frailty or trajectory of frailty between the haemodialysis and peritoneal dialysis groups. This is in keeping with a study by Iyasere et al. in prevalent haemodialysis and peritoneal dialysis patients, in which no difference in physical function was detected between the groups.

We identify presence of frailty before the initiation of KRT as an important predictor of increased hospitilisation (a marker of increased morbidity) after starting KRT. The association of frailty with hospitilisation and mortality in patients on KRT is well-described. Johansen et al. and McAdams-DeMarco et al. both reported an association between frailty and mortality and hospitilisation in prevalent haemodialysis patients^5,8^, whilst Kamijo et al. has described increased risk of death in frail patients receiving peritoneal dialysis^9^. Additional studies have observed that the frailty score at the time of referral to a pre-dialysis clinic^21^ and at dialysis initiation were both associated with increased mortality^7,10^. These studies have all been based on assessment of frailty at a single time point. Our work builds on this existing evidence by demonstrating that trajectory of frailty progression before and after starting KRT may be associated with increased hospitilisation and risk of death. We identify a deterioration in frailty status in the first 6 months of dialysis treatment as a predictor of morbidity and mortality; highlighting a period of vulnerability in the patient journey and a potential window for intervention. This underscores the importance of repeated frailty assessments in the early months of dialysis. Our data suggests that those with an increase in CFS in this period should be identified as at-risk.

This study included a large and ethnically diverse cohort of incident dialysis patients. It benefits from the inclusion of 102 peritoneal dialysis patients, a larger sample than has been reported in the majority of the existing evidence. The haemodialysis and peritoneal dialysis groups were similar in age, degrees of comorbidity and baseline frailty score and we were therefore able to draw meaningful comparisons between the two cohorts. As discussed, frailty trajectory has not been directly compared between the two modalities in previous literature.

A major limitation of this retrospective study is missing data. Nephrology staff at our center are encouraged to record CFS as part of routine clinical care, however, the timing and frequency of recordings are subject to clinical judgement and not protocolized. This results in variability between patients and explains the missing data in this study. It is possible that more frail patients or those with a clinically apparent change in frailty would have more frequently recorded frailty scores than non-frail or stable patients. Our analysis shows that those with complete data are older than the group with missing data, however, the groups are otherwise similar in their characteristics and mean CFS at each timepoint. Sensitivity analysis using multiple imputation does suggest that our findings are robust to missing data under the assumption that observations are missing at random. Nevertheless, this highlights a major challenge in working with routine data and a need for future prospective studies in order to further our understanding of frailty trajectory on KRT.

A further limitation is that there may be some variation in the application of the CFS between clinical staff. Given that CFS was recorded as part of routine clinical care and data collected retrospectively, the inter-rater reliability in our cohort is not known. Previous studies have reported good inter-rater reliability (cohens κ 0.74) when the CFS is used in acute settings (e.g. emergency departments)^22^. There is limited data in chronic dialysis populations. We were only able to access admissions records at our centre, and associate hospital in our sector of North London. We cannot exclude the possibility of admissions to other hospitals, as we do not have routine access to this data. However, the likelihood is low due to local policy and non-availability of dialysis facilities at neighbouring centers. Finally, level of available social support may be an important modifying factor of both frailty trajectory and hospitilisation. We were unable to capture this data retrospectively, however, understanding the impact of social support on outcomes in this population should be a focus of further research.

In conclusion, we report no improvement in frailty after initiation of KRT and the presence of frailty prior to dialysis was associated with increased hospitilisations. These findings underline the importance of pre-dialysis frailty screening in order to inform prognosis and patient counselling in ESKD. Our data suggests that those with a CFS ≥ 5 are at increased risk and would benefit from early identification. We identify deterioration in frailty status over the first 6 months of dialysis treatment as a predictor of both increased hospitalisations and mortality. These findings highlight the utility of serial frailty measurements in the early months of dialysis treatment as a clinical tool for identification of at-risk patients and prognostication and a trigger for intervention to preserve physical functioning.

## Supplementary Information

Below is the link to the electronic supplementary material.

Supplementary Material 1

## Data Availability

Data was collected as part of clinical audit by members of the direct care team and is not available to be shared. However, data are available from the corresponding author on reasonable request.
