# Supplementary material for: Trajectory of frailty and association with adverse outcomes in patients with end-stage kidney disease over the first year of dialysis
Source: Sci Rep. 2025 Oct 8;15:35092. doi: 10.1038/s41598-025-18961-4 (PMC12508114; doi:10.1038/s41598-025-18961-4)
Supplement: Supplementary file 1 — Supplementary Material 1 [file 41598_2025_18961_MOESM1_ESM.docx]

| **Supplementary Table 1: Mean baseline clinical frailty score (CFS) by age group (n = 189)** | | | | |
| --- | --- | --- | --- | --- |
| **Age group** | **n** | **Mean baseline CFS** | **Standard deviation** | **Oneway ANOVA p-value** |
| **< 55** | 27 | 3.62 | 1.84 | 0.087 |
| **55 to 64** | 34 | 3.76 | 1.56 |  |
| **65 to 74** | 59 | 4.07 | 1.46 |  |
| **≥ 75** | 69 | 4.38 | 1.33 |  |

| **Supplementary Table 2: Mixed effects linear regression model for the association of mean clinical frailty score (CFS) with time on dialysis (multiple imputation models)** | | | | | | |
| --- | --- | --- | --- | --- | --- | --- |
|  | **Model adjusted for age, sex, Charlson Comorbidity Index (CCI) and dialysis modality**  **(n = 293)**  **10 imputations** | | **Model adjusted for age, sex, Charlson Comorbidity Index (CCI) and dialysis modality**  **(n = 293)**  **40 imputations** | | **Model adjusted for age, sex, Charlson Comorbidity Index (CCI) and dialysis modality**  **(n = 293)**  **80 imputations** | |
|  | **Effect estimate** | **95% confidence interval** | **Effect estimate** | **95% confidence interval** | **Effect estimate** | **95% confidence interval** |
| **Mean change in CFS per 6 months on dialysis** | 0.23 | 0.10 – 0.36 | 0.23 | 0.10 – 0.36 | 0.23 | 0.11 – 0.36 |
